# Polysaccharide-Based Edible Biopolymer-Based Coatings for Fruit Preservation: A Review

**DOI:** 10.3390/foods13101529

**Published:** 2024-05-14

**Authors:** Athira R. S. Pillai, Ansu Sara Eapen, Wanli Zhang, Swarup Roy

**Affiliations:** 1 Department of Food Technology and Nutrition, School of Agriculture, Lovely Professional University, Phagwara 144411, Punjab, India; athirapillai99@gmail.com (A.R.S.P.); ansuangelin@gmail.com (A.S.E.); 2 School of Food Science and Engineering, Hainan University, Haikou 570228, China

**Keywords:** edible coating, polysaccharide, fruit preservation, fruit quality, shelf life, safety

## Abstract

Over the last decades, a significant rise in fruit consumption has been noticed as they contain numerous nutritional components, which has led to the rise in fruit production globally. However, fruits are highly liable to spoilage in nature and remain vulnerable to losses during the storage and preservation stages. Therefore, it is crucial to enhance the storage life and safeness of fruits for the consumers. To keep up the grade and prolong storage duration, various techniques are employed in the food sector. Among these, biopolymer coatings have gained widespread acceptance due to their improved characteristics and ideal substitution for synthetic polymer coatings. As there is concern regarding the safety of the consumers and sustainability, edible coatings have become a selective substitution for nurturing fruit quality and preventing decay. The application of polysaccharide-based edible coatings offers a versatile solution to prevent the passage of moisture, gases, and pathogens, which are considered major threats to fruit deterioration. Different polysaccharide substances such as chitin, pectin, carrageenan, cellulose, starch, etc., are extensively used for preparing edible coatings for a wide array of fruits. The implementation of coatings provides better preservation of the fruits such as mango, strawberry, pineapple, apple, etc. Furthermore, the inclusion of functional ingredients, including polyphenols, natural antioxidants, antimicrobials, and bio-nanomaterials, into the edible coating solution matrix adds to the nutritional, functional, and sensory attributes of the fruits. The blending of essential oil and active agents in polysaccharide-based coatings prevents the growth of food-borne pathogens and enhances the storage life of the pineapple, also improving the preservation of strawberries and mangoes. This paper aims to provide collective data regarding the utilization of polysaccharide-based edible coatings concerning their characteristics and advancements for fruit preservation.

## 1. Introduction

Currently, there has been a huge demand for the production of fruits globally due to their importance in human consumption. Fruits such as mango [1], apple [2], banana [3], raspberry, and strawberry [4] serve as a rich source of nutrients that can provide enormous health benefits to consumers and prevent chronic diseases. Apple contains a high number of polyphenols, pectin, and dietary fibers that are particularly effective for preventing cardiovascular diseases and diabetes and contribute to antioxidant effects due to the presence of polyphenols [2]. Similarly, mangoes are efficient for their gastroprotection and anti-bacterial effects [1]. It is expected that there will be a 60 percent rise in production by 2050 [5]. Besides production, storage of the produce is also a major challenge, that is, the prevention of postharvest loss, which is considered a major hindrance to food and nutritional security. This can cause excessive waste production, which can be a threat to the ecosystem that limits the availability during the off-season. It has been reported that at the postharvest stage, around 40 percent of fresh produce is lost; this was observed mainly in developing nations. India holds the second position in the production of fruits and vegetables after China, where 70 percent of the production is subjected to loss due to inadequate storage conditions [6]. 

Preserving fruits is vital for multiple reasons. The preservation process of nutrients ensures that consumers can still access the benefits of fruits even when they are not freshly harvested. Moreover, preservation enables year-round availability of fruits, irrespective of seasonal variations in production, which is specifically advantageous for areas where certain fruits are not cultivated or are only available in particular seasons [7]. When it considers an economic perspective, fruit preservation creates opportunities for farmers to sell their produce beyond the harvest season and fosters employment in processing and packaging industries related to fruit preservation. As fruits can be consumed in raw form and do not require any processing methods, the importance of storing fruits without delaying or compromising their freshness is very important for consumer acceptance [8].

Improper harvesting techniques, handling, and storage conditions can promote microbial proliferation and significantly hasten the degradation and fruit spoilage. Abrasions and cuts formed during harvesting and transportation can facilitate the entry of microbes through the fruit surface, pose a persistent threat, and cause rapid deterioration. Furthermore, chemical changes such as oxidation and browning can also occur when the fruits are not stored in appropriate environmental conditions [9]. Methods such as canning, drying, and freezing can cause nutritional loss, which further reduces acceptability and market demand [10]. While cold storage has been deemed an effective method for fruit preservation, it has induced negative effects via chilling injury, leading to the loss of fruit texture, flavor, and color, which ultimately diminishes the market value and consumer acceptance of the affected fruits. Packaging is considered a better choice for fruit preservation by providing structural protection from contamination elements like gas, moisture, pathogens, and light. Due to rising concerns regarding the ecosystem and health, the utilization of synthetic packaging films/coatings or chemical-based treatments is not recommended at all. The methods, including modified atmospheric packaging and wax coating on fruit surfaces, can be used for fruit storage [11]. Modified atmospheric conditions provide an environmental condition that delay the oxygen level inside the packaging condition, thereby reducing the ripening. Controlling the oxygen level in the modified atmospheric packaging can often promote the expansion of anaerobic pathogens on the fruit surface, and similarly, toxic chemicals such as morpholine were added to waxes for modifying coating properties and are found to have carcinogenic effects [12]. Edible coatings or preserving fruits with polymer from natural sources has occupied wide popularity due to their potential benefits and, moreover, can be consumed safely along with the fruits [13,14].

Coating edible materials is emerging as a promising replacement for prolonging the storage duration of fruits by preventing deterioration. Most quality losses of fresh produce occur during storage and transport, including escape of moisture, which causes fruit shrinkage, reduction in weight, microbial attack, chemical degradation, etc. The coating surface of fruit restricts free gas transfer and prevents unwanted odors [15]. These coating solutions are formulated from renewable sources and are coated as a narrow layer on the fruit surface to elevate the storage duration by minimizing the moisture loss and acting as a barrier of gas, microbes, and light, thus slowing down the decaying process. As edible coatings are prepared from living sources, they are a superior substitute for synthetic or petroleum-based coatings, which can pose a threat to the environment [16]. Synthetic polymers such as polypropylene, polystyrene, polyethylene, and polyolefin can accumulate as microplastic in surroundings and result in bioaccumulation and the production of harmful volatile compounds (acrolein and propanal) via photo-degradation [17]. The production of biopolymers is expected to increase by 2.41 million tons by 2030 [18]. Polysaccharides such as cellulose, pectin, chitin, starch, and alginate are broadly used for the synthesis of coatings. Utilizing polysaccharides for edible coatings presents numerous advantages, as they are sourced from plants, algae, or via microbial fermentation and offer a sustainable and eco-friendly solution due to their inherent biodegradability [19]. By forming a protective barrier against gases and external factors, they exhibit high structural integrity; thus, they effectively extend the life duration of fruits, ensuring their freshness and quality over time. Moreover, polysaccharides are widely acknowledged as being safe for direct food contact by regulatory authorities, enhancing their selectivity for various implementations in the food industry. Moreover, reducing the reliance on traditional packaging materials and covering them with edible substances contributes to the reduction in packaging waste, aligning with sustainable practices and environmental conservation efforts. Additionally, certain polysaccharides, such as dietary fibers, may offer potential health benefits, including improved digestive health and cholesterol management, further adding to their appeal in food applications [20].

The inclusion of natural substances like herb extract or essential oils into the edible coating can provide additional benefits as they serve as natural antimicrobials and antioxidants for consumers. These substances contain ample active substances such as polyphenols, flavonoids, and terpenoids, which can prevent oxidation and browning reactions in fruits, the major attribute for fruit deterioration, and are recognized as safe to consume [21]. Furthermore, bio-nanomaterials like cellulose nanofillers [22], chitosan nanocrystals [23], and starch nanoparticles [24] also attained much popularity in the packaging industry due to their divergent characteristics when utilized in edible coatings. Several researchers found that the implementation of bio-nanomaterials as a filler in the edible coating of fruits such as grapes [25], mango [26], strawberries [27], etc., can modify the edible coating characteristics and enhance the fruit storage duration.

There are several research studies conducted to evaluate the application of polysaccharide-based edible coating for maintaining the quality and storage life of different fruits [28,29,30]. Numerous reviews are also available on polysaccharide-based edible coatings for fruits, vegetables, meat, and other foods. However, specific reviews on the polysaccharide edible coatings, particularly for fruits, are limited. This review emphasizes the overview of several analyses and research studies focused on the use of polysaccharide-based edible coating and its prospects for maintaining fruit quality and as a sustainable method for fruit preservation. The review gathers information regarding innovative coating materials, methods, their practical applications, and recommendations for improving the characteristics and limitations of polysaccharide edible coatings for enhancing fruit preservation. This includes compiling the recent investigations on the efficiency of polysaccharide edible coatings blended with active substances and bio-nanomaterials in extending the shelf life of fruits via different mechanisms such as inhibiting microbial proliferation, controlling moisture mitigation, and controlling browning reactions for sustaining quality attributes of different fruits. 

## 2. Fruit Preservation Techniques and Their Limitations

### 2.1. Physical Preservation

At present, the commonly used methods of postharvest fruit preservation are mainly physical treatments, such as temperature regulation, humidity regulation, controlled atmosphere treatment, and radiation treatment [31]. Low-temperature storage is the most common postharvest physical preservation method for fruits. At low temperatures, the rate of physiological metabolism of the fruit is significantly reduced, thus maintaining a high commercial value. However, some tropical and subtropical fruits are susceptible to chilling injury at low temperatures, which can cause physiological disorders. Similarly, fruit metabolism can be controlled by regulating the humidity of the environment. In addition, radiation treatments, including ultraviolet radiation, are commonly used for postharvest physical storage of fruits to improve fruit quality, mainly via the mechanisms of direct sterilization and induction of fruit resistance [32]. In addition, controlled atmosphere treatments are also common physical preservation treatments that regulate the aging process of fruits by changing the ratio of carbon dioxide to oxygen in the environment, thereby directly affecting the respiratory metabolism of the fruits. Generally speaking, as the proportion of carbon dioxide increases, the proportion of aerobic respiration of the fruit decreases, but too low a proportion of oxygen will cause anaerobic Shanghai to the fruit. Therefore, the key to gas conditioning treatment is the need to find the right proportion of gas to minimize the outdated respiration trumpet and not produce low oxygen injury [33].

It is worth noting that the main principle of edible coated films as a green fruit preservation method is to reduce gas exchange with the fruit in the external environment by forming a semi-permeable coating film on the surface of the fruit. Therefore, the edible coating is also a physical preservation method and is similar to micro-gas conditioning [34]. The application of edible coatings in fruit preservation has its origins in the waxing technique. Waxes used for fruit coating are synthesized from petroleum or natural sources or esters derived from monohydroxy alcohols and carboxylic acid with high molecular weight. The coating acts as a better prevention for oxygen, moisture, and microbes and imparts a shiny aspect to the fruits, enhancing consumer preference. A thin sheet of coating is applied on fruits such as apples, oranges, grapes, avocados, watermelons, muskmelons, etc. The application of heat treatment can cause whitish–waxy formation on the fruit surface [35]. The waxy coating has several limitations over edible coating. Mainly, these disadvantages depend on the concentration, thickness, and preservatives added to the wax to modify the coating properties [36]. Morpholine is considered a highly hazardous chemical added to fruit wax as an emulsifier. Regular consumption of morpholine can cause damage to internal organs. Furthermore, it can have carcinogenic effects, through the formation of N-nitroso morpholine via nitrosation [12]. Therefore, edible films/coatings containing natural additives can be considered possible alternatives for protecting fruits that are safe to consume along with the fruits. These coatings can maintain fruit quality and sensory characteristics and prevent chemical reactions such as browning and oxidation, adding benefits to the consumers as a source of natural, free radical scavengers and microbe inhibitors. Waxes derived from plant sources such as rice wax, which is separated from rice bran, are found to be suitable for coating and can supply nutritional components and are approved by the Food and Drug Administration [37]. 

### 2.2. Chemical Preservation

In addition to the physical preservation methods mentioned above, the postharvest preservation of fruit also relies heavily on several chemical preservatives [38]. These chemical preservatives are mainly fungicides and plant hormones. Chemical fungicides have strong antifungal activity and can inhibit the growth of various fungi on the surface of the fruit, thus directly slowing down the occurrence of fruit rot. The other types of chemical preservatives are mainly plant hormones or plant hormones, which can directly regulate the process of fruit ripening and aging [39]. At present, the most common fruit postharvest preservative is 1-MCP, which is a plant ethylene inhibitor and can be used as an ethylene receptor to inhibit the production of ethylene in the fruit. Whereas ethylene is considered to be an important signaling molecule in inducing fruit ripening and senescence, 1-MCP slows down the fruit senescence process by inhibiting the production of fruit ethylene. Similarly, some other chemo insulators belong to endogenous hormones, e.g., melatonin, salicylic acid, and methyl jasmonate. In addition, some plant extracts, such as phenolics, have also been reported to be effective in delaying postharvest fruit senescence [32].

## 3. Different Polysaccharide-Based Edible Coatings for Fruit Preservation

### 3.1. Cellulose

Cellulose is one of the plenteous biodegradable polymers, obtained from the membrane of plant cells or algae using is widely used for the synthesis of coating preparation due to its versatile properties. It consists of glucose monomers linked by β-1,4 bond. The distinct property of cellulose includes the ability to withstand high mechanical stress and temperature, making it a chosen alternative for plastic packaging [40]. One of the challenges exhibited by cellulose is its high hydrophilicity, which restricts the resistance to moisture in the food system when it is applied as a coating substance. This drawback can be overcome by the blending of active or hydrophobic substances or the inclusion of nanofillers into the cellulose matrix. The surface coating of curcumin-added chitosan/cellulose nanofiller-based coating films significantly preserved kiwi’s quality for up to 10 days. Curcumin and chitosan are proven the prevent the growth of pathogens, and along with cellulose nanofillers, further attributed to the prevention of weight loss and the ripening process of kiwi, thereby enhancing the storage time and physiological characteristics of the fruit [41].

### 3.2. Pectin

Pectin is one of the complex polysaccharides, which is predominantly found in plants with high molecular weight and branched structure consisting of β-(1,4,)-D- galacturonic acid can be considered the suitable polysaccharide for the production of fruit coatings or composite film due to its potential to scavenge free radicals [42]. They provide significant resistance to moisture and gases, and highly transparent can contribute to maintaining fruit preservation by maintaining its sensory and quality parameters. Edible films/coatings synthesized using pectin successfully enhanced the storage time for various fruits such as plums [43], sapota [44], mango [14], strawberries [45], etc. The surface coating made out of pectin for plum storage lessens the activity of the oxidative enzyme polyphenol oxidase and enhances the activity of the antioxidant enzyme peroxidase, resulting in improved antioxidant activity and superior quality of the plum during storage [44]. Similarly, the coating of a formulation consisting of pectin, bee wax, monoglyceride, and glycerol reduced the physiological and chemical changes in mango during storage, which favors maintaining fruit quality attributes for 13 days [46].

### 3.3. Starch

Starch, which is categorized as a polysaccharide, is a hydrocolloid polymer composed of amylopectin and amylose obtained from cereals or tubers which is widely used for preservation due to its high oxygen-resisting capacity, transparency, and easy availability at less cost. Amylose is a linear structure consisting of α-1,4 bonds, whereas amylopectin is a branched polysaccharide containing α-1,6 bonds. Upon the inclusion of plasticizers, starch transforms into thermoplastic, making it unique from other polysaccharides. Mostly these plasticizers are sugars (fructose) or polyols (glycerol, sorbitol) obtained from waste [47]. The drawback exhibited by starch is its high hydrophilicity nature which affects the moisture resistance. This can be rectified by mixing with co-polymer or the inclusion of natural additives [48]. The implementation of starch-gelatin film/coating on avocado fruit enhanced the storage time of the fruit by reducing the respiration rate and observed improved firmness and color retention [49]. 

Starch–chitosan application on the surface of papaya significantly affected its storage time and preserving qualities. Surface coating controlled the synthesis of volatile compounds such as ethyl butanoate, butyric acid, and ethyl hexanoate, thereby delaying papaya fermentation during storage. Another major factor causing papaya deterioration is microbes, where coated papaya prevents the growth of microorganisms while storage time compared to uncoated fruit [50]. Starch-based film coating on strawberries prevented microbial contamination, particularly fungal infection. The addition of plasticizer and concentration of amylose has a notable influence on maintaining the strawberry shelf period. As the quantity of amylose increased there was a formation of the compact structure resulting in the reduction in moisture permeability. Furthermore, plasticizers interact with amylose and result in high mechanical characteristics [51]. Corn starch and chitosan nanoparticles with thymol increased the shelf life of cherry tomatoes as they provided high antioxidant and antimicrobial properties [52]. The shelf life of fresh-cut pear was increased by the antioxidant and antimicrobial starch film incorporated with *Adiantum capillus* extract [53].

### 3.4. Chitosan

Chitosan, synthesized primarily from the shell of crustaceans (shrimp and crabs) as well as certain fungi and insects, constitutes a polysaccharide characterized by a molecular structure comprising glucosamine and N-acetylglucosamine units. In food preservation, chitosan serves as a versatile edible coating with multiple functionalities. Its applications are diverse, ranging from forming a protective barrier on food surfaces to reducing moisture loss, exchange of gases, and microbial incidence. Moreover, its inherent antimicrobial properties enable chitosan to combat a broad spectrum of microorganisms, such as bacteria, fungi, and yeast, thereby restricting spoilage and mitigating health risks associated with foodborne pathogens [54]. 

### 3.5. Carrageenan

Natural polymer carrageenan is synthesized from red algae (*Eucheuma spinosum* and *Eucheuma cottonii)* via isolation or hydrolysis methods. The structure of carrageenan consists of disaccharide units 3,6-anhydrous-D-galactopyranose and D-galactopyranose linked with α-1,3 and β-1,4 bonds. Due to its biodegradability and ability to be obtained easily, carrageenan is utilized for the synthesis of film/coating in the food industry. The property of carrageenan can be enhanced by adding components while used as an edible coating [55]. 

Carrageenan and green tea extract have been utilized as coatings for blueberries and raspberries [56], while alginate–oleic acid-based coatings supplemented with green tea have been applied to strawberries and raspberries [57]. These coatings offer antiviral activity, contributing to their preservation and quality enhancement. The edible coating is prepared by using polysaccharide carrageenan and carboxy methyl cellulose effectively increasing the storage of tomatoes by 12 days compared to control tomatoes. These films significantly prevented decay and moisture loss and maintained the physio-biochemical and nutritional properties of the fruit [14]. 

Similarly, the active component from pomegranate incorporated alginate/agar film increased the preservation period and quality parameters of the fig including high firmness, antioxidant, and mechanical properties. The coating of the surface resisted the transfer of moisture from the fruit to the surroundings, thereby reducing the transpiration rate and resulting in control of weight loss [58].

### 3.6. Alginate

Alginate is a natural polysaccharide extracted from brown seaweed and is extensively used for coating in fruits owing to its colloidal property characterized by the composition of mannuronic acid and guluronic acid units. Alginate is coated successfully on various fruits like plums [59] and guava [60] and preserved fruit quality over the storage period. Utilization of alginate along with chitosan blended with black cumin extract enlarged the storage period of guava and prevented microbial contamination [60].

### 3.7. Pullulan

Pullulan is mainly obtained via the fermentation of certain fungi and yeast. Its structure is composed of maltotriose units linked by α-1,6 glycosidic bonds and is highly soluble in hot and cold water [61]. The coating can form a transparent and flexible covering and is effective in resisting moisture and gas, which degrade fruits. The use of a pullulan and chitosan-combined coating on mango was efficient in maintaining the fruit’s freshness [62].

### 3.8. Natural Gum

**Figure 1:**
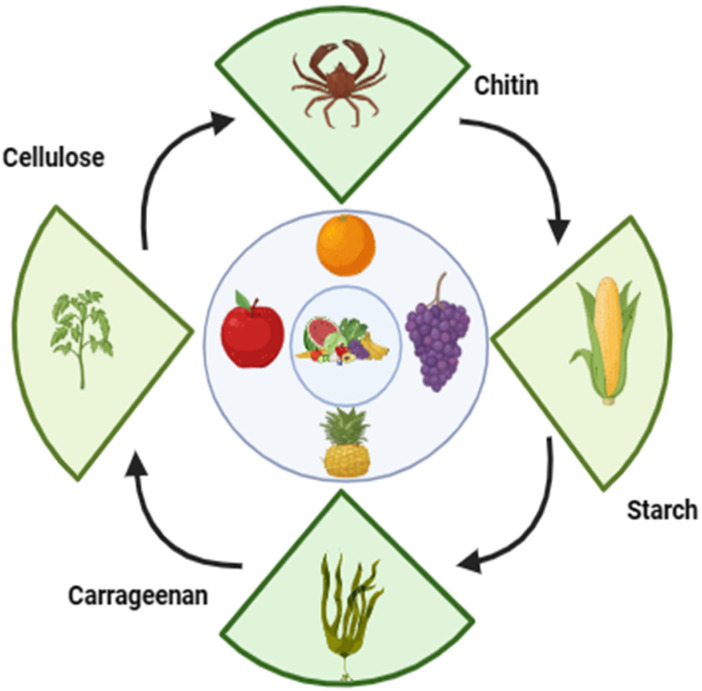
Sources of different polysaccharides for edible coating.

Gums are complex polysaccharide compounds that exhibit the unique ability to transform into a gel-like substance when combined with water, sourced predominantly from wood barks or seed coatings [63]. Their distinctive properties, characterized by a lack of toxicity and high biocompatibility, have positioned them as valuable materials for various applications, particularly in fruit preservation as coating material. Given their natural origin and compatibility with biological systems, gums have found significant potential as edible coatings for fruits, offering a safe and effective means of extending shelf life and maintaining freshness [64]. The gums are primarily classified into gum Arabic, tara gum, xanthan gum, guar gum, Tragacanth gum, locus bean gum, and Persina gum, etc., based on the source of origin. Guar gum is most commonly used as an edible coating because of its distinctive characteristics contributed by high molecular weight. It has been reported that the incorporation of mint extract (10%) and citric acid into guar gum prevented the browning reaction in the ber fruit and prolonged the shelf life compared to the control fruit [65]. Different types of polysaccharides, their sources, and their characteristics are illustrated in Table 1 and Figure 1.

### 3.9. Use of Biopolymer Nanostructure for the Preparation of Edible Coating

In food packaging, nanomaterials have acquired a significant position because of their enormous beneficial properties over conventional packaging and improvement in the characteristics of natural coatings. Nanomaterials are defined as substance sizes varying from 1 to 100 nm [66]. Nanomaterials are incorporated with biopolymers to form homogenous dissemination, thereby modifying the edible coatings in terms of biodegradability, mechanical strength, barrier, and optical characteristics. Nanomaterial, which is added into the matrix cross-links with the biopolymer, prevents the interaction of moisture and gas entry into the food and provides high mechanical strength via strong bond formation [67]. The major concern related to the application of nanotechnology is its impact on human health. Some research has shown that nanomaterials can cause the formation of oxidative stress. To overcome this, bio-nanomaterials can be chosen as a superior alternative [68]. Nanomaterials prepared from plant sources such as cellulose, chitin nanoparticles, and starch nanomaterials are widely utilized as an edible coating due to their advanced benefits and sustainability [66].

Recently, there has been a drastic increase in the application of bio-nanofillers for the preservation of fruits via coating. Bio-nanomaterials as edible coating maintain temperature stability and modify the barrier characteristics of the film and can be considered a better option for elevating the storage duration of fruits after harvest without compromising their quality [23]. Coatings of cellulose–nanofiber emulsions on the surface of the banana had a remarkable influence in preventing the ripening and extending the storage period. The high crystalline nature and aspect ratio of cellulose nanocrystals make it ideal for the preparation of edible coating. In addition to providing high resistance to gas and moisture, and mechanical strength, the coating controlled ethylene production and ensured fruit quality [22]. Chitosan nanomaterials were applied to the edible coating to preserve the quality of grapes from microbial contamination. This was because of the high charge density along with the high surface area of chitosan nanoparticles’ destruction of the negatively charged microbial cell wall [25].

**Table 1:** Various polysaccharides, their sources, and characteristics.

Polysaccharide Based Biopolymer	Sources	Advantages	Disadvantages	Refs.
Cellulose	Plant cell wall and algal cell wall	Abundant in nature, Increased water holding capacity, High mechanical strength, non-toxicity, high crystalline property, and high molecular weight	High water absorption capacity reduces the water resistance in packaging	[69]
Chitosan	Insect exoskeleton and crustaceans	High antimicrobial activity, antioxidant and pigment absorption, biocompatible	Solubility in aqueous solution is poor	[54]
Alginate	Algae	Biocompatibility, High structural integrity and long-term storage capacity, thickening capacity, emulsifier, and stabilizer	Limitation in moisture barrier property, unpleasant odor, and cause precipitation at less pH	[70]
Starch	Plant sources such as cereals and potatoes, cassava	Reduced cost, biodegradable and abundant in nature, high mechanical property, selective permeability to gases	Requires plasticizers to improve the adhesion property	[71]
Pullulan	Fungal source	Barrier to oxygen and high thermal stability, good structural flexibility, water-soluble, high adhesion property	High cost, breakability, and high hydrophilicity	[72]
Carrageenan	Extracted from red seaweed	Biocompatibility and bio-adhesives	High hydrophilicity and poor mechanical strength	[73]
Pectin	Dicotyledonous plants and fruit peel like apple	Gel formation, biodegradability, emulsifier, and prebiotic properties	Hydrophilicity	[74,75]
Natural gum	Seeds and guar	Techno-functional properties, biocompatibility, thickening agent, and emulsifier	Limitation in mechanical and structural characteristics in raw form	[76,77]

## 4. Techniques for the Synthesis of Biopolymer-Based Edible Coatings for Fruit Preservation

### 4.1. Dipping Method

Dipping is the immersion of fresh fruits into an edible coating solution for a specific period. The second step is deposition, wherein the coating adheres to the fruit surface, resulting in the formation of a thin protective coating that resists the contaminants and factors that cause fruit degradation. Any excess amounts of liquid or solvent on the coating are removed via evaporation, forming a stable solid thin layer [78]. Factors such as viscosity, density, and surface tension of the solution influence the thickness of the film. The major limitation of dipping is a non-uniform coating over the surface, which can cause inadequate coating of particular fruits, which can affect the fruit freshness [79].

### 4.2. Layer-by-Layer Edible Coating

The layer-by-layer edible coating is an emerging method that provides multiple layers on the surface for fruit preservation. The method involves the combination of two or more differently charged biopolymer coating via electrostatic deposition. This technique is an excellent alternative to the monolayer method which limits adhesion to the fruit surface [80]. Furthermore, the layer-by-layer method attributed to better resistance of gases exhibited high antioxidant and antimicrobial characteristics and potential control of ethylene production and retained fruit quality. Edible coatings made out of alginate and chitosan triple- and penta-layer-by-layer coatings limited the production of ethylene hormone in Japanese pear which enhanced the fruit freshness and increased the storage time [81]. Similarly, the deposition of polysaccharide films made of chitosan and carboxymethyl cellulose restored the fruit quality of strawberries in terms of firmness and aroma components, thereby having a significant role in maintaining the strawberry quality after harvest [82].

### 4.3. Spraying Method

**Figure 2:**
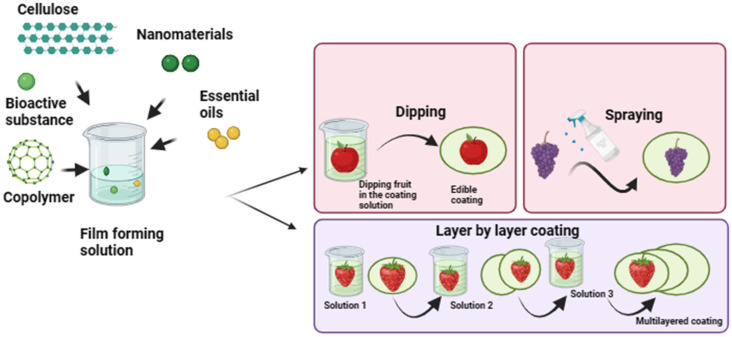
Different techniques are involved in the preparation of edible coatings.

The spraying technique for edible coatings entails the coating of biopolymer substance onto the food surface via a specialized spraying method. As an initial step, an edible coating solution is prepared by various edible substances cellulose or chitosan, and suitable additives into a mixture [83]. To attain proper atomization and uniform surface coverage, the viscosity of the solution is adjusted to the desired level by altering the concentration of the added substances or thickeners. A spray gun or airbrush is operated for the spraying process by loading the solution into it. This step is followed by drying to obtain a stable uniform coating over the surface of fruit. These techniques can be further divided into air-spray atomization (which utilizes air-surrounded fluid with low speed), air-assisted airless atomization, and pressure atomization (air is replaced by pressure). An edible coating synthesized from cellulose nanofiber applied on the grape surface via the spraying technique maintained freshness by preventing moisture transfer and high mechanical characteristics, unlike uncoated grapes [84]. Different techniques for coating preparation are illustrated in Figure 2.

### 4.4. Panning Method

The panning technique for coatings consists of tumbling the food product to be coated in a large bowl. Then, the suitable solution is dusted onto the surface of the fruit by spinning the pan and forced air is applied to dry the coated solution at a higher temperature. This method offers a versatile solution for surface coating and is highly suitable for industrial scale-up, especially round-shaped fruits [85].

### 4.5. Fluidized Bed Coating

The fluidized bed coating technique is currently used in the food industry for coating substances on food materials but has comparatively less acceptance due to the high cost and requirement of a huge quantity of coating solutions. Fluidized bed coating is further divided into top spray and rotating fluidized bed bottom spray, and the top spray is found to be more efficient. The method involves the use of a nozzle to spray the fruit surface at low pressure. The method can prevent the formation of clusters, which is a major problem with the panning method and requires less time for the coating process [86].

## 5. Properties of Polysaccharide-Based Edible Coatings for Fruit Preservation

**Figure 3:**
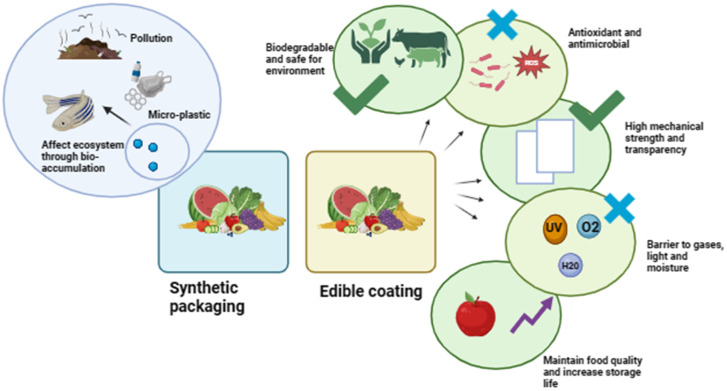
Different characteristics of the edible coating.

Various physical and functional properties of polysaccharide-based edible coating applicable for fruit preservation are discussed in this section. The various important characteristics of edible coatings are represented in Figure 3.

### 5.1. Barrier Properties

For the extended life span and preservation of fruits, the most important property should an edible coating is the ability to act as an obstacle that affects the fruit quality. Barrier properties of edible coatings refer to their capacity to limit the passage of gases, moisture, light, and other harmful elements that could compromise the integrity of the product; biopolymer-based coatings aim to preserve or even enhance these properties while minimizing their environmental impact. This is achieved via various means, such as careful material selection, the use of multilayer structures, the application of barrier coatings, and the adoption of advanced technologies [87]. Via the optimization of these strategies, coatings can effectively protect fruits from external factors transmission, while simultaneously reducing their ecological footprint. Additionally, innovations involve incorporating or blending natural substances such as herb extracts, bio nanocomposites, or essential oils, which can effectively obstruct gas and water vapor transmission [88].

The polar nature of polysaccharides hinders the permeability of non-polar gas oxygen, which is a major factor that significantly contributes to food deterioration via oxidation. Furthermore, they can alter the other quality parameters, including organoleptic and nutritional composition. One limitation exhibited by polysaccharide coatings is their moisture resistance; this can be overcome by the inclusion of natural additives [89]. The starch–carrageenan coating infused with fatty acid on plum fruit inhibited the permission of carbon dioxide, oxygen, and ethylene synthesis. According to the result, the firmness and phytochemical remained unchanged on coated plums and the storage period was extended without losing fruit quality [90]. 

### 5.2. Optical Properties

The optical attributes of coatings play a crucial role in both functionality and aesthetic appeal, with sustainable materials being selected based on their transparency, translucency, or opacity to suit the specific needs of the packaged product and consumer preferences; for example, transparency facilitates product visibility, enhances consumer experience, and enables viewing of package contents, while controlled light transmission properties not only showcase products attractively but also safeguard against harmful ultraviolet radiation, thereby extending shelf life, whereas controlled opacity can provide privacy and protection for sensitive items while maintaining an appealing appearance [91]. The significance of optical properties in the conception and execution of sustainable packaging solutions is underscored by the delicate equilibrium between transparency, protection, and aesthetic allure. Recently, there has been a notable surge in enhancing biopolymer materials by incorporating natural substances or bio-nanomaterials to modify the optical attributes of films [92].

### 5.3. Structural Properties

The composition of edible films/coatings plays a pivotal role in determining their structural properties. Recycled plastics contribute durability and effective barrier properties, whereas bio-based polymers offer remarkable strength and flexibility [93]. By judiciously selecting and blending these components, we can form films/coatings customized to suit diverse product requirements and application scenarios. Moreover, to enhance the performance of such films or coating, supplementary substances like UV stabilizers, antioxidants, and antibacterial agents can be incorporated [94].

Carbohydrates, which serve as the fundamental constituents of edible-based films/coatings, are primarily sourced from natural reservoirs such as microorganisms and plants due to their complex structural characteristics. Cellulose, starch, chitosan, gum Arabic, agar, and their derivatives stand out as the most commonly utilized polysaccharides in packaging [95]. These polymers exhibit significant potential in crafting a diverse array of edible and non-edible covering, owing to their unique properties. Polysaccharide-based films/coatings have been thoroughly investigated for their ability to mitigate the detrimental effects of oxidation, dehydration, rancidity, surface browning, and oil diffusion, thereby enhancing the physicochemical, nutritional, and sensory attributes of food products while concurrently extending their shelf life. Notably, chitosan, derived from chitin, boasts a semi-crystalline structure and demonstrates the capacity to yield films/coatings endowed with enhanced mechanical strength and antibacterial effect [29]. Gum Arabic, a complex polysaccharide, exhibits the ability to form amorphous films characterized by favorable flexibility and moisture barrier properties. Meanwhile, agar, comprising a blend of polysaccharides, agarose and agaropectin, finds application in sustainable packaging endeavors owing to its remarkable gelation and thickening capabilities [94].

The structure, arrangement, and properties of amylose and amylopectin polymers within starch molecules, as well as the distribution of crystalline and amorphous regions within starch granules, play a significant role in determining the structural characteristics of starch-based films/coatings [95]. The presence of hydroxyl groups in polysaccharide-based films/coatings influences their structural attributes, leading to increased intermolecular interactions, greater crystallinity, and higher melting temperatures.

### 5.4. Thermal Properties

Thermal stability serves as a crucial parameter in preventing substance degradation due to temperature fluctuations, ensuring packaging materials retain their properties during transportation, storage, and production stages [96]. Biodegradable packaging or edible coatings materials derived from renewable sources, like natural polymers or recycled materials, are engineered to uphold integrity and functionality even under elevated temperature conditions. Maintaining the thermal resilience of biopolymers is essential to avert adverse outcomes such as warping and chemical leaching, which could compromise food quality and safety [97].

During coating/film development, a prevalent approach involves a blend of active substances into biopolymers to enhance coating properties. The effect of polysaccharide-based functional coatings in fruit preservation is discussed in Table 2. Notably, the strategic combination of cellulose and chitosan has been demonstrated to effectively support temperature stability, thereby aligning with sustainable packaging objectives. The incorporation of lignin-containing nanocellulose, together with chitosan, facilitates the formation of covalent bonds between lignin and the polymer matrix. This interaction contributes to enhanced thermal resistance within the packaging coating or film [98]. Recently there has been a new approach for improving thermal stability coating via irradiation. A composite film consisting of polyvinyl alcohol/carboxymethylcellulose/tannin irradiated with gamma rays prolonged the storage time of a banana to 19 days without losing its quality. The high energy radiation promoted the compatibilization between the polymers, leading to the enhanced modification of the film in terms of thermal and mechanical stability [99]. 

### 5.5. Antimicrobial Properties

Microbes contribute to a large extent to the postharvest loss of fruits. Bacteria and fungi are prominent in the development of postharvest diseases, which severely affect the fruit quality. Mechanical damage results in wounds or bruises during harvesting, leading to the penetration of microbes, and the storage condition can promote microbial deterioration. The use of chemical-based pesticides has been adopted widely for prevention which can cause severe health issues to the consumer [111]. Coating the fruit surface with edible materials is found to be a better alternative to this and can prevent the entry of moisture and gases and enhance the characteristics that influence the storage life. Furthermore, natural additives are added to replace synthetic antimicrobials such as triclosan, which can adversely affect human health [112]. Active substances derived from plant sources, like phytochemicals and essential oils, are considered safe to use and can be successfully incorporated into the coating film instead of direct application to food due to their sensory attributes [113]. The research conducted by [114] coating the surface of mango with pectin and oregano essential oil successfully suppressed the growth of *Salmonella enterica* and *Colletotrichum gleosporioides,* which cause anthracnose disease, a major postharvest disease in mango. 

This substance directly interacts with the cell wall of microorganisms thereby disrupting the membrane resulting in the loss of electrolytes or organelles of microbes. Another mechanism involves the inducement of reactive oxygen species, which leads to cell damage and death of the organism [115,116]. Several researchers contributed to the addition of natural additives into the biopolymer edible coating for protecting the fruits. An investigation conducted by the authors of [117] discovered the antimicrobial effect of chitosan/pullulan film blended with a pomegranate peel extract, which retained the freshness of litchi. The inclusion of nanoemulsion of citral component into sodium-alginate edible coating positively prevented the foodborne pathogen *Salmonella enterica* and *Listeria monocytogenes* and improved the freshness of pineapple [118]. 

### 5.6. Antioxidant Properties

The impact of fruit deterioration is reliant on the concentration of reactive oxygen species; at a lower level, it can prevent the growth of harmful pathogens, whereas a higher concentration significantly destroys the fruit’s firmness, freshness, and loss of several beneficial nutrients. Nutrients, especially vitamin C, are degraded rapidly after harvest. After harvest fruits lack their natural defense mechanism against oxidative stress. Climatic fruit’s continuous respiration process, even after harvesting, results in an oxidation reaction via the excess absorption of oxygen. Fruits such as avocado, which contain high lipids, are more prone to oxidation reactions resulting in flavor in fruits. To prevent this, one possible method is to use antioxidants incorporated into the packaging system. Antioxidants act as radical scavengers by donating free electrons and neutralizing the unpaired reactive oxygen species. Furthermore, antioxidants can provide an immune system via the regulation of antioxidant enzymes, including catalase and superoxide dismutase, which detoxify free radicals. Butylated hydroxy anisole, ethoxyquin, and butylated hydroxytoluene are chemical-based antioxidants. Based on the dosage, individual susceptibility, and other external factors can lead these synthetic antioxidants to harmful effects. Therefore, natural antioxidants can be incorporated into edible films for sustainable and healthy usage. Furthermore, they can provide additional benefits to the coating as several properties and health benefits to the consumer. The chitosan-based edible coating has significantly influenced the antioxidant effect in fruits such as strawberries [23], peaches [119], figs [56], kiwi [55], and papaya fruits [51]. The addition of phytochemicals such as polyphenols, flavonoids, and terpenoids in the form of extract or powder into the biopolymer is highly recommended because they can further increase the antioxidant potential of the film. The research found that chitosan film induced the production of the antioxidant enzyme NADH oxidase and superoxide dismutase, thereby regulating oxidative stress in wounded apples [120]. Aloe vera gel coating also greatly influenced the activity of superoxide dismutase and catalase for maintaining freshness and prevention of oxidational damage of coated guava during storage [121].

### 5.7. Enzymatic Browning

Enzymatic browning in fruits is a phenomenon that causes brown color formation in horticultural products when subjected to mechanical damage, cutting, or bruising due to a natural chemical process carried out by enzymes, most notably polyphenol oxidase. The reaction initiates when the cut portion of the fruit is exposed to oxygen, leading to the oxidation of phenolic compounds, causing a brownish appearance in fruits like apples and bananas. Adoption of suitable methods for the prevention of browning in fruits is crucial to enhance the shelf life and prevent food waste accumulation. Dipping the fruits in a solution containing anti-browning agents such as glutathione, citric acid, and ascorbic acid is found to be useful in inhibiting the polyphenol oxidase (PPO) activity by reducing the pH condition, where PPO is active at optimum pH 5–7. However, health concerns related to the chemical substance and less consumer preference towards synthetic additives limit their usage in the food industry. The most effective anti-browning agent, sulfates, was banned by the Food and Drug Administration because of side effects [122]. The edible coating containing natural active substances is a better choice for consumers which is safe and sustainable and acts as a good carrier of anti-browning agents. Compounds that possess antioxidant activity are highly prone to suppress browning reactions by interacting with the intermediate products and preventing the formation of melanin pigment [123,124].

A protective food coating was prepared using hydroxypropyl methylcellulose incorporated with lemon essential oil, and aloe vera gel is highly effective for preventing browning in apples. Limonene and ocimene are the major substances present in lemon that can provide nutritional benefits and are Generally Recognized As Safe (GRAS). Furthermore, several studies proved the antimicrobial properties of aloe vera and their addition to biofilm can modify the characteristics of the edible film. Apple is highly susceptible to browning, which reduces its nutritional quality and can be successfully prevented via the hydroxy propyl methyl cellulose coating containing aloe vera–lemon essential oil additives [125].

Like apples, bananas are also highly vulnerable to browning reactions. Recent research regarding the prevention of peel browning and chilling injury in bananas was successful by using astragalus polysaccharide, which has high phytochemical activities such as antioxidant, anti-inflammatory, and anti-diabetic. The film has a significant effect on preventing the accumulation of hydrogen peroxide and enhances the activity of antioxidant enzymes superoxide dismutase and catalase, leading to the preservation of fruit nutrients [126].

### 5.8. Adhesion

Adhesion refers to the force of attraction between two different substances, like the fruit surface and coating material. The coating should strongly adhere to the fruit surface, otherwise, it can result in the easy peel-off of the substance and allow the permeability of gases, moisture, and other contaminants. In addition, the adhesion ensures uniform distribution of the coating for maintaining fruit freshness and quality [127]. The selection of suitable polysaccharide material is very important for abiding adhesion in the edible coating. There should be less variation between the surface energy of the coating material and the surface energy of the substance to be coated to increase the adhesion [128]. Certain polysaccharide substances limit adhesion properties and binding between the coating and the fruit surface and form less interaction. The high hydrophilic nature of polysaccharides possesses low adhesion. Molecular weight, degree of substitution and concentration, and compatibility with fruit surface are considered for the enhancement of adhesion. To overcome this limitation, functional compounds incorporated into the biofilm were adopted [129]. Physical inclusion can affect stability and also cause loss of substance via evaporation which can result in less stability. This can be altered by adopting various feasible techniques for the addition and coating of materials. By adopting the mussel-adhesion technique, chitosan is functionalized with catechol, significantly modifying the film/coating attachment to the fruit surface of bananas and strawberries [130]. Covalent grafting of epigallocatechin (EGCG) with low methoxy pectin (LMP) coatings on grapes reduced weight loss and improved adhesion. The pyrogallol structure of EGCG contributed to the enhancement of the adhesion and wetting properties of the pectin to the fruit surface via strong bond formation and crosslinking [131]. 

### 5.9. Fruits’ Firmness and Texture

Firmness is a significant quality attribute that contributes to consumer acceptability. After harvest, fruits are subjected to a series of physiological transformations impacted by factors such as emission of ethylene, rate of respiration, and enzymatic activities, all of which contribute to variation in fruit firmness and texture. These differences encompass the degradation of cell walls, reduction in turgor pressure, and enzymatic breakdown of crucial structural components like pectin, cellulose, and hemicellulose, resulting in a continuous softening and loss of crispness in fruits over time [132]. Polysaccharide-based edible coatings emerge as an essential component in preserving fruit firmness and texture by retarding the rate of physiological processes responsible for softening. Additionally, these coatings exhibit high antimicrobial properties, effectively preventing microbial proliferation and decay, thus extending the fruits’ shelf life. Furthermore, polysaccharide coatings serve as structural reinforcements, bolstering the integrity of the cell wall and mitigating mechanical damage during handling and transportation [93]. Consequently, by safeguarding the structural composition of fruits, polysaccharide coatings play a pivotal role in enhancing the overall quality, ensuring that consumers receive fruits with optimal firmness and texture characteristics. Fruits such as strawberry, cherry, pear, and litchi are highly prone to the loss of firmness after harvest. Studies revealed that the protective layer made of chitin, carboxy methyl cellulose, Arabic gum, and alginate has a positive attribute on preserving fruit firmness and maintaining fruit quality over the period [117,132,133,134].

## 6. Constraints of Utilizing Polysaccharide Edible Coating

Even though polysaccharides have immense advantages as an edible coating due to biocompatibility and biodegradability, they exhibit certain limitations based on the type of polysaccharide. These limitations are the key to their practical utilization in fruit preservation. For example, chitosan is effective in providing an antimicrobial effect, but solubility in neutral and alkaline pH conditions is very low [54]. Starch is a widely available polysaccharide that requires a plasticizer to modify its adhesion to fruit surface and structural characteristics [49]. Cellulose, the most abundant polysaccharide, provides less resistance to moisture conditions [69]. Alginate is popular for its gelling property, which can produce an unpleasant odor and precipitate in acidic conditions [70]. To overcome this limitation, a combination of biopolymer or blending with natural additives like herbs containing bioactive substances can be effectively used to modify the coating properties. Chitosan incorporated with cinnamon oil maintained the quality of the pineapple and prevented *E. coli* growth for 11 days [100]. Black cumin extract infused with alginate edible coatings maintained the firmness of the guava and reduced the weight loss and respiration rate compared to the control guava [60]. Therefore, the fabrication of functional polysaccharide-based blend coatings can be advantageous over simple coatings to develop effective preservation and improvement in the shelf life of fruits.

## 7. Conclusions

In conclusion, polysaccharide-based edible coatings offer a promising substitute to synthetic polymer packaging substances for preserving fruits for a long time due to their biodegradability and sustainability and the generally recognized as safe (GRAS) status, elevating their application as an edible coating. The coating can effectively delay fruit respiration, moisture loss, and microbial entry, maintain fruit firmness, texture, color, and sensory parameters, and reduce the reliance on synthetic polymers, which can pose a threat to the environment. Studies have shown that polysaccharide substances, particularly chitosan, can delay the growth of disease-causing pathogens and preserve fruit quality. From various types of research, it is suggested to use biopolymer coating formulations blended with natural copolymer, bio-nanomaterials, and phytochemicals to provide additional advantages by modifying the properties such as barrier, mechanical, and thermal and attribute to its nutritional characteristics. Natural antioxidants and antimicrobials obtained from herbal sources increased the functionality of the coating and consumer demand for edible coatings. In short, polysaccharide composites incorporated with natural bioactive elements are a better option for protecting fruits from degradation and can be considered a sustainable method to prevent postharvest loss. Even though there is potential in biopolymer-based coatings, there are still challenges associated with this method, such as high hydrophilicity, cost, etc., which need to be improved, and thus, further research on this topic could be beneficial for practical fruit preservation applications. Future studies have to be conducted to evaluate the bioavailability and release of nutrients in polysaccharide edible coatings and the optimization of coating formulations to achieve the desired balance between various properties of the coating. Moreover, the stability and functionality of the coating also should be studied elaborately for multiple fruits. Investigations and surveys can be conducted to understand the consumer perception of edible coatings to ensure market adoption and the development of innovative methods that are economically feasible for scaling up the production of edible coatings for large-scale fruit preservation operations. By addressing these recommendations, polysaccharide-based edible coatings can become a major solution for preserving fruits, contributing to a more sustainable and environmentally friendly food packaging industry. 

## Figures and Tables

**Table 2:** Effect of various polysaccharide coatings on the shelf life of fruits.

Edible Coating	Active Ingredient	Fruit Preserved	Key Results	Shelf Life	Refs.
Chitosan	Apple peel polyphenols	Strawberry	Prevented fruit decay, maintained total phenol content, firmness, and anthocyanin, and reduced weight loss Pros and cons: High antimicrobial properties but limits in solubility	Increase in shelf life	[29]
Chitosan	Cinnamon essential oils	Pineapple	Antimicrobial activity *Escherichia coli* and *Salmonella* spp. maintained fruit firmness and prevented weight loss Pros and cons: High barrier property and prevent microbe proliferation, but limits against oxygen barrier	Increased shelf life up to 11 days	[100]
Carboxymethyl cellulose and pectin		Plum	Maintained firmness, reduction in weight loss titratable acidity, vitamin C, flavonoid, and antioxidant activity Pros and cons: High mechanical strength but causes high moisture permeability	Increase in shelf life	[101]
Carboxymethylcellulose	Aloe vera	Apple	Prevented weight loss, and microbial growth, browning, increase in titratable acidity Pros and cons: Abundant in nature but limited in barrier properties and flexibility	Increased shelf life up to 10 days	[102]
Carrageenan	Green tea extract	Raspberries and blueberries	Anti-viral activity against murine norovirus and hepatitis A virus, preservation of firmness Pros and cons: High adhesiveness, good gelling properties but poor flexibility	Increase in shelf life	[56]
Chitosan and starch		Papaya	Maintained firmness, reduced weight loss, and microbial growth Pros and cons: Chitosan is effective in preventing microbes, and starch has good mechanical strength, but chitosan limits solubility, and starch is susceptible to recrystallization	Increased shelf life by 15 days	[50]
Pectin		Plum	Reduced polyphenol oxidase activity, maintained polyphenol content and anthocyanin and antioxidant capacity Pros and cons: Good gel-forming properties and transparency but lack in stability at particular temperature conditions	Increase in shelf life	[43]
Chitosan/pullulan	Pomegranate peel extract	Mango	Increase in fruit firmness, texture, antioxidant activity, TSS, and reduced weight loss Pros and cons: Pullulan is highly transparent, excellent oxygen barrier material but has limited availability and is highly expensive	Increase in shelf life 18 days	[62]
Gum		Peach	Retarded ethylene production, weight loss, softening of fruit, and maintained nutritional content Pros and cons: Excellent thickening agent, but an excess amount can cause a gummy texture	Increased storage time by maintaining the quality of the peach fruit	[64]
Starch	Cellulose nanofibers and basil essential oil	Mandarin orange	Prevented weight loss and maintained fruit color Pros and cons: Highly accessible and cost-effective but limits in stability and barrier properties	Increased storage life for 12 days	[103]
Carboxymethyl cellulose/chitosan/Polyvinyl alcohol	Nano curcumin	Sweet orange	Maintained fruit freshness, reduced weight loss, and antimicrobial properties against *Bacillus subtills*, *Staphylococcus aureus* and *Escherichia coli* Pros and cons: Polyvinyl alcohol is transparent and has good film-forming properties but is highly water-sensible	Increased storage life for 56 days	[104]
Hydroxy methyl cellulose and sodium alginate	Asparagus extract	Strawberry	Antifungal against *Penicillium italicum*, reduced weight loss, and increased phenol and flavonoid content Pros and cons: Sodium alginate has excellent gelling properties, and hydroxymethyl cellulose is transparent but lacks stability and mechanical properties	Increase in shelf life	[105]
Alginate	Black cumin extract	Guava	Antibacterial against *Staphylococcus hominis* and *Escherichia coli*, reduced respiration rate and weight loss, retained vitamin C, phenols, and flavonoids Pros and cons: High thermal resistance and gel-forming properties but can interact with other ingredients	Increase in shelf life	[60]
Xanthan	Cinnamic acid	Pears	Inhibit the activity of browning enzymessuch as peroxidases (POD) and polyphenol oxidase (PPO), prevent the oxidation of phenols into melanincompounds Pros and cons: Good adhesion and flexibility but can affect sensorial characteristics of food	Increased storage time by maintaining the quality	[106]
Starch	Whey protein	Plum	Reduce the respiration rate and weight loss retention. Pros and cons: Flexible and transparent but has a poor barrier and mechanical properties, also Whey protein is expensive	Increased shelf life	[107]
Zein	Resveratrol	Apple slices	Reduced moisture loss and increased color retention Pros and cons: Forms a glossy appearance, but an excess amount can cause brittle and sensitivity to pH	Increased storage time by quality retention and nutrient delivery	[108]
Chitosan	Vanillin, cinnamaldehyde, and mandarin extract	Melon	Antimicrobial activity, good sensory quality, maintained fruit quality Pros and cons: Strong and flexible but can cause allergy due to shellfish in certain people	Increased shelf-life by enhanced quality and sensory properties	[109]
Zein	Argentinian propolis extracts	Raspberries	Maintained fruit quality, freshness, firmness, and antimicrobial activity Pros and cons: Highly transparent and glossy appearance but can cause allergy problems	Increased shelf life up to 11 days	[110]

## Data Availability

No new data were created or analyzed in this study. Data sharing is not applicable to this article.
